# Transcriptional analysis of *Amorphotheca resinae* ZN1 on biological degradation of furfural and 5-hydroxymethylfurfural derived from lignocellulose pretreatment

**DOI:** 10.1186/s13068-015-0323-y

**Published:** 2015-09-04

**Authors:** Xia Wang, Qiuqiang Gao, Jie Bao

**Affiliations:** State Key Laboratory of Bioreactor Engineering, East China University of Science and Technology, 130 Meilong Road, Shanghai, 200237 China

**Keywords:** Furfural, 5-Hydromethylfurfural (HMF), *Amorphotheca resinae* ZN1, Biodetoxification, Transcription level, qRT-PCR

## Abstract

**Background:**

Furfural and 5-hydroxymethylfurfural (HMF) are the two major inhibitor compounds generated from lignocellulose pretreatment, especially for dilute acid, steam explosion, neutral hot water pretreatment methods. The two inhibitors severely inhibit the cell growth and metabolism of fermenting strains in the consequent bioconversion step. The biodetoxification strain *Amorphotheca resinae* ZN1 has demonstrated its extraordinary capacity of fast and complete degradation of furfural and HMF into corresponding alcohol and acid forms. The elucidation of degradation metabolism of *A*. *resinae* ZN1 at molecular level will facilitate the detoxification of the pretreated lignocellulose biomass and provide the metabolic pathway information for more powerful biodetoxification strain development.

**Results:**

*Amorphotheca resinae* ZN1 was able to use furfural or HMF as the sole carbon source for cell growth. During the detoxification process, *A. resinae* ZN1 firstly reduced furfural or HMF into furfuryl alcohol or HMF alcohol, and then oxidized into furoic acid or HMF acid through furan aldehyde as the intermediate at low concentration level. The cell mass measurement suggested that furfural was more toxic to *A. resinae* ZN1 than HMF. In order to identify the degradation mechanism of *A*. *resinae* ZN1, transcription levels of 137 putative genes involved in the degradation of furfural and HMF in *A. resinae* ZN1 were investigated using the real-time quantitative PCR (qRT-PCR) method under the stress of furfural and HMF, as well as the stress of their secondary metabolites, furfuryl alcohol and HMF alcohol. Two Zn-dependent alcohol dehydrogenase genes and five *AKR*/*ARI* genes were found to be responsible for the furfural and HMF conversion to their corresponding alcohols. For the conversion of the two furan alcohols to the corresponding acids, three propanol-preferring alcohol dehydrogenase genes, one NAD(P)^+^-depending aldehyde dehydrogenase gene, or two oxidase genes with free oxygen as the substrate were identified under aerobic condition.

**Conclusions:**

The genes responsible for the furfural and HMF degradation to the corresponding alcohols and acids in *A*. *resinae* ZN1 were identified based on the analysis of the genome annotation, the gene transcription data and the inhibitor conversion results. These genetic resources provided the important information for understanding the mechanism of furfural and HMF degradation and modification of high tolerant strains used for biorefinery processing.

**Electronic supplementary material:**

The online version of this article (doi:10.1186/s13068-015-0323-y) contains supplementary material, which is available to authorized users.

## Background

Pretreatment is the key step to overcome the recalcitrance of lignocellulosic biomass for subsequent enzymatic hydrolysis and microbial fermentation [1]. In this process, various inhibitory compounds to hydrolytic enzymes and fermenting strains are generated due to the partial over-degradation of lignocellulose, such as furan derivatives, weak organic acids and phenolic compounds [2–6]. Among these inhibitors, two furan aldehydes, furfural and 5-hydroxymethylfurfural (HMF) derived from the dehydration of pentose and hexose are the strongest inhibitors owing to the abundance and strong toxicity to microorganisms [7, 8]. To remove the inhibitors from the pretreated lignocellulose (“detoxification”), water washing, overliming, ion exchange absorption, solvents extraction and other methods have been tested but massive waste water generation, solids material loss, and high processing cost are frequently occurred [9, 10].

**Fig. 1:**
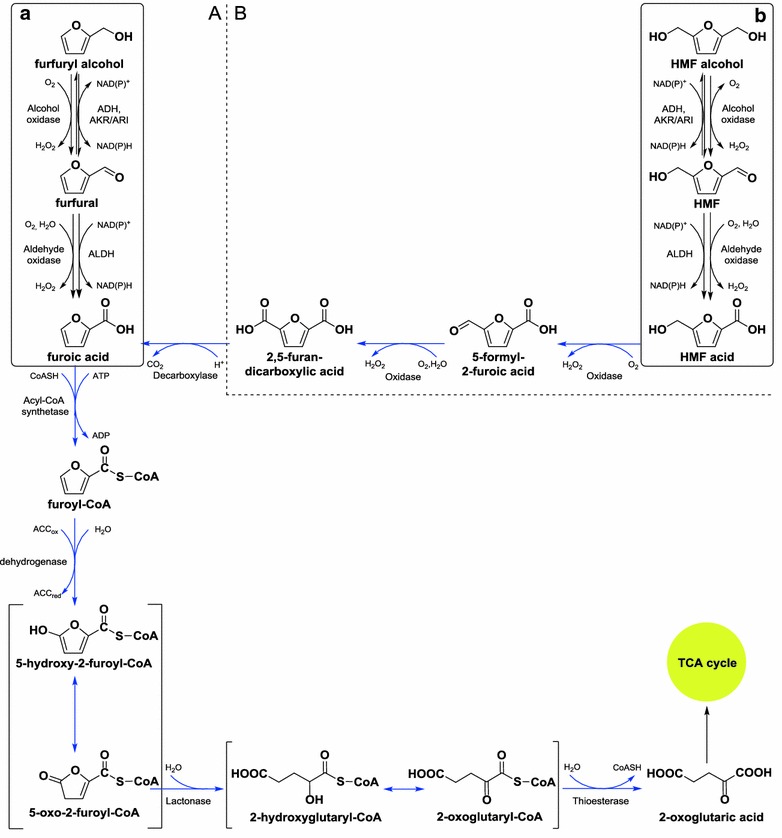
Metabolic pathways of furfural (*A*) and HMF (*B*) degradation in *A. resinae* ZN1. *Solid boxes* (**a**, **b**) were based on the previous experimental phenomena [24]. *Blue arrows* were adapted from Trudgill [18] and Koopman et al. [20]. ACC, acceptor, either oxidized (ox) or reduced (*red*). *ADH* alcohol dehydrogenase, *AKR* aldo–keto reductase,* ARI* aldehyde reductse, *ALDH* aldehyde dehydrogenase

In recent few years, a biological detoxification method using specific microorganisms to convert furfural and HMF into non-toxic substances was proposed and the method demonstrated the unique advantages such as mild condition, low energy demand and no waste water generation [11–13]. Many biodetoxification microorganisms have been discovered and the biodetoxification mechanisms were extensively investigated [14–17]. Trudgill [18] proposed a putative degradation pathway of furfural in *Pseudomonas putida* F2 in 1969, and then verified by Koenig and Andreesen [19] and Koopman et al. [20]. Koopman et al. [20] extended the pathway to HMF in *Cupriavidus basilensis* HMF14. Zhang et al. isolated a kerosene fungus *Amorphotheca resinae* ZN1 [21] with fast and complete biodetoxification of almost all toxic inhibitors and has been practically applied for the high performance of ethanol, lipid, and lactic acid production [21–23]. The degradation performance of furfural and HMF by *A. resinae* ZN1 was investigated and a hypothesized metabolic pathway was illustrated in Fig. 1 in the previous studies [21, 24]. Furfural is quickly reduced to furfuryl alcohol, then re-oxidized into its aldehyde form (furfural) again but at a much lower and harmless concentration then oxidized into its acid form (furoic acid) under aerobic condition; furoic acid is subsequently ligated coenzyme-A into furoyl-CoA, hydroxylated into α-oxoglutaric acid and CoA, and finally α-oxoglutaric acid is metabolized via tricarboxylic acid cycle (TCA) (Fig. 1a). Similar to furfural, HMF is quickly reduced to HMF alcohol, re-oxidized to the aldehyde (HMF) under aerobic condition, then oxidized to its monocarboxylic acid (5-hydroxymethyl-furoic acid, HMF acid) and dicarboxylic acid (2, 5-furandicarboxylic acid, FDCA); FDCA is converted into furoic acid via a decarboxylation reaction, and joins into furfural catabolism (Fig. 1b). The first several steps (Fig. 1a, b) from furfural (HMF) to furfuryl alcohol (HMF alcohol) or furoic acid (HMF acid) demonstrated the primary detoxification function in *A*. *resinae* ZN1 because the two metabolites are less toxic or even non-toxic to fermenting strains [25, 26].

Currently, the metabolic pathways of furfural and HMF degradation by the mentioned biodetoxification strains are still a putative picture. The best acknowledged pathways of furfural and HMF in *C*. *basilensis* HMF14 were investigated by transposon mutant screening, but the complete gene information responsible for the furfural or HMF degradation had not been fully identified due to the limitation of the method used [20]. In this study, *A. resinae* ZN1 was cultured in flasks under the stress of furan aldehyde inhibitors allowing cell biomass measurement and subsequent cell samples collection, then the inhibitor degradation pathways in *A. resinae* ZN1 were constructed and the genes involved in the degradation of furfural and HMF were investigated based on the whole genome annotation information, the inhibitor conversion experimental results, and the relevant studies. The transcription and expression levels of the genes were analyzed using the real-time quantitative PCR (qRT-PCR) method under the stress of furfural and HMF, as well as the stress of the secondary metabolites furfuryl alcohol and HMF alcohol. The key genes responsible for the furfural and HMF degradation to furan alcohols and furan acids in *A. resinae* ZN1 were identified. This study enables us to understand the furfural and HMF degradation mechanism in the practically applied biodetoxification strain *A. resinae* ZN1 at the genetic level and facilitates the future metabolic engineering of more powerful biodetoxification strains.

## Results and discussion

### Cell growth and degradation metabolism under the stress of furfural and HMF

**Fig. 2:**
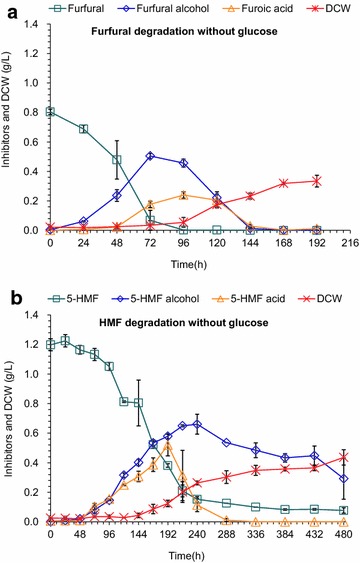
Degradation of furfural (**a**) or HMF (**b**) without glucose by *A. resinae* ZN1. Conditions: inoculum 10 % (v/v), 28 °C, natural pH in static state culture. Mean values are presented with *error bars* representing two standard deviations

The cell growth and degradation metabolic performance of *A. resinae* ZN1 were investigated when furfural or HMF was used as the sole carbon source (Fig. 2). Figure 2a shows that furfural was completely utilized within 96 h, and then the cell mass growth started to quickly increase. Furfuryl alcohol and furoic acid increased with the decrease of furfural, then decreased from their maxima and were completely utilized after 144 h. Figure 2b shows that HMF was degraded in a similar way to furfural when HMF was used as the sole carbon source but with a much lower rate. The cell mass quickly increased when only half of the initial HMF was consumed, comparing to the cell mass increasing from almost zero furfural existence. Figure 2 suggests that both furfural and HMF were able to be used as the sole carbon source for the cell growth of *A. resinae* ZN1, and furfural was more toxic to *A. resinae* ZN1 than HMF.

**Fig. 3:**
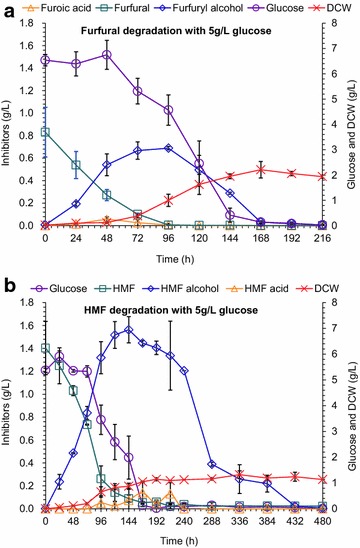
Degradation of furfural (**a**) or HMF (**b**) with 5 g/L of glucose by *A. resinae* ZN1. Conditions: inoculum 10 % (v/v), 28 °C, natural pH in static state culture. Mean values are presented with *error bars* representing two standard deviations

To accumulate enough cell mass of *A. resinae* ZN1 for qRT-PCR test, glucose was added as the carbon source together with furfural or HMF. Figure 3 shows that furfural and HMF were converted into furfuryl alcohol and HMF alcohol, respectively, but the glucose consumption and cell growth were very slow until furfural and HMF were decreased to a low level. The results indicate that the two furan aldehydes were prior to glucose as the substrates for *A. resinae* ZN1. Figure 3 also reveals the difference of furfural and HMF degradation in *A. resinae* ZN1 when glucose was added. For furfural, the presence of glucose did not affect the degradation rate of furfural to furfuryl alcohol, but the degradation of furfuryl alcohol to furoic acid was prolonged. The cell mass was increased to 2 g/L (Fig. 3a), approximately one order of magnitude greater than that without glucose addition (0.3 g/L). It was also observed that furoic acid concentration was very low although furfuryl alcohol was quickly decreased with glucose addition, indicating that furoic acid was metabolized quickly after its formation into the central carbon metabolism. For HMF, the presence of glucose surprisingly accelerated the conversion of HMF to HMF alcohol and the decrease of HMF approximately was equal to the increase of HMF alcohol (Fig. 3b). The conversion of HMF alcohol to HMF acid was accelerated only when glucose was almost degraded completely. The maximum cell mass with glucose existence was 1.2 g/L, almost threefolds greater than that without glucose addition.

The degradation results illustrate that the degradation pathway of *A. resinae* ZN1 started with the quick degradation of furfural and HMF into their less toxic metabolites, furfuryl alcohol and HMF alcohol, then into furoic acid and HMF acid, respectively; finally the two furan acids were metabolized completely into the central carbon metabolism. The existence of glucose did not affect the degradation rate of furfural to furfuryl alcohol, but promoted the degradation rate of HMF to HMF alcohol. The existence of glucose inhibited the further conversion of the two furan alcohols into the corresponding acids, especially for HMF alcohol conversion. Under the stress of furfural and HMF, *A. resinae* ZN1 reduced the two furan aldehydes into furfuryl alcohol and HMF alcohol firstly, and then oxidized to their corresponding acids (furoic acid and HMF acid) through the formation of aldehyde intermediates at low concentration levels. The three consequent conversion reactions composed the essential steps of furfural and HMF degradation in *A. resinae* ZN1.

### Selection of putative genes responsible for degradation of furfural and HMF

The genes responsible for the degradation of furfural and HMF to the less toxic compounds include the NAD(P)H- or NAD(P)^+^-dependent alcohol dehydrogenases (ADH), aldo–keto reductases/aldehyde reductases (AKR/ARI), and aldehyde dehydrogenases (ALDH) as illustrated in Fig. 1. Furfural (HMF) is reduced quickly, aerobically or anaerobically, into furfuryl alcohol (HMF alcohol) by NAD(P)H dependent alcohol dehydrogenases (ADH) or aldo–keto reductases/aldehyde reductases (AKR/ARI) with the consumption of NAD(P)H. In the following aerobic oxidation steps, the genes responsible for the oxidation of furfuryl alcohol (HMF alcohol) to furfural (HMF) at much lower concentration include ADH genes or AKR/ARI genes with the formation of NAD(P)H, as well as aldehyde dehydrogenases (ALDH) genes with the formation of NAD(P)H responsible for the further oxidation into furoic acid (HMF acid). Under anaerobic condition, the degradation of furan aldehydes stops at furan alcohols without furan acids formation [24]. Thus in these steps, the alcohol oxidases and/or aldehyde oxidases genes with free oxygen as substrate for the oxidation of furan alcohols into furan acids were also included.

To provide the strong evidence for the degradation pathways of furfural and HMF in *A. resinae* ZN1, the relevant genes encoding the enzymes on the furfural and HMF degradation pathways were thoroughly screened and selected from the whole genome of *A. resinae* ZN1 (GenBank: JZSE00000000). Then, the transcription levels of these selected genes under the stress of furfural and HMF, as well as the stress of their derivatives furfuryl alcohol and HMF alcohol were examined using qRT-PCR. The relevant genes encoded four categories of enzymes, including alcohol dehydrogenases (ADH), aldo–keto reductases/aldehyde reductases (AKR/ARI), aldehyde dehydrogenases (ALDH), and oxidases as shown in Table 1. Overall, total 137 relevant genes with the putative functions on furfural and HMF degradation were screened from the genome of *A. resinae* ZN1 considering the functional possibilities.

**Table 1:** Genes involved in furfural and HMF degradation in *A. resinae* ZN1

Functional categories	Annotation description	No.	Gene symbols
Furan aldehydes to furan alcohols and vise verse			
Alcohol dehydrogenase (ADH, totally 80 genes)	Zn-dependent alcohol dehydrogenase	27	Arz_10052_T1; Arz_10290_T1; Arz_1137_T1; Arz_13167_T1; Arz_13908_T1; Arz_1429_T1; Arz_1478_T1; Arz_15335_T1; Arz_1542_T1; Arz_15626_T1; Arz_15717_T1; Arz_15727_T1; Arz_15928_T1; Arz_16054_T1; Arz_16075_T1; Arz_1653_T1; Arz_16562_T1; Arz_17261_T1; Arz_17817_T1; Arz_4514_T1; Arz_4549_T1; Arz_5226_T1; Arz_6335_T1; Arz_9116_T1; Arz_92_T1; Arz_9386_T1; Arz_9803_T1
	Short-chain dehydrogenase	31	Arz_10032_T1; Arz_10048_T1; Arz_10445_T1; Arz_10735_T1; Arz_11749_T1; Arz_12708_T1; Arz_12851_T1; Arz_13165_T1; Arz_14225_T1; Arz_14914_T1; Arz_15221_T1; Arz_16631_T1; Arz_17974_T1; Arz_18688_T1; Arz_2180_T1; Arz_3303_T1; Arz_3412_T1; Arz_5014_T1; Arz_5127_T1; Arz_5257_T1; Arz_5925_T1; Arz_6148_T1; Arz_6276_T1; Arz_6334_T1; Arz_6568_T1; Arz_6769_T1; Arz_7751_T1; Arz_8436_T1; Arz_9070_T1; Arz_9496_T1; Arz_9792_T1
	Alcohol dehydrogenase, iron-type	2	Arz_5124_T1; Arz_962_T1
	Other alcohol dehydrogenase	20	Arz_11219_T1; Arz_11558_T1; Arz_1162_T1; Arz_12683_T1; Arz_12736_T1; Arz_12928_T1; Arz_15224_T1; Arz_15907_T1; Arz_15995_T1; Arz_17851_T1; Arz_18719_T1; Arz_18811_T1; Arz_2579_T1; Arz_3164_T1; Arz_3236_T1; Arz_3617_T1; Arz_6090_T1; Arz_6576_T1; Arz_6619_T1; Arz_9528_T1
Aldehyde reductase, aldo/keto reductase (AKR/ARI, totally 21 genes)	Aldo/keto reductase	12	Arz_10923_T1; Arz_13395_T1; Arz_14857_T1; Arz_14938_T1; Arz_1621_T1; Arz_16490_T1; Arz_17182_T1; Arz_17370_T1; Arz_17920_T1; Arz_3860_T1; Arz_7295_T1; Arz_8147_T1
	Aldehyde reductase	9	Arz_13663_T1; Arz_137_T1; Arz_15150_T1; Arz_18349_T1; Arz_3141_T1; Arz_3976_T1; Arz_7271_T1; Arz_7657_T1; Arz_8367_T1
Furan aldehydes to furan acids			
Aldehyde dehydrogenase (ALDH, totally 20 genes)	NAD-dependent aldehyde dehydrogenase	15	Arz_10708_T1; Arz_11689_T1; Arz_12503_T1; Arz_15082_T1; Arz_1535_T1; Arz_15746_T1; Arz_18373_T1; Arz_3957_T1; Arz_494_T1; Arz_5090_T1; Arz_5413_T1; Arz_6133_T1; Arz_7774_T1; Arz_9159_T1; Arz_9778_T1
	Aminoadipate-semialdehyde dehydrogenase	1	Arz_11723_T1
	Salicylaldehyde dehydrogenase	2	Arz_18463_T1; Arz_3707_T1
	Betaine aldehyde dehydrogenase	1	Arz_10838_T1
	Semialdehyde dehydrogenase	1	Arz_9969_T1
Oxidase (totally 16 genes)	Alcohol oxidase	5	Arz_11534_T1; Arz_14616_T1; Arz_17610_T1; Arz_5225_T1; Arz_6129_T1
	Glucose oxidase	2	Arz_16765_T1; Arz_18116_T1
	Choline oxidase	2	Arz_10839_T1; Arz_12679_T1
	Cholesterol oxidase	1	Arz_17625_T1
	Glyoxal oxidase	2	Arz_15963_T1; Arz_3499_T1
	Ent-kaurene oxidase	4	Arz_16317_T1; Arz_17995_T1; Arz_18300_T1; Arz_6529_T1

Totally 80 *ADH* genes screened from the genome of *A. resinae* ZN1 were hypothesized to be responsible for furfural and HMF degradation, including 27 Zn-dependent alcohol dehydrogenases, 31 short-chain dehydrogenases, 2 iron-dependent alcohol dehydrogenases. Besides the genome annotation for alcohol dehydrogenases genes, 20 genes with the action site on the CH-OH group were also selected such as arabinitol dehydrogenase, butanediol dehydrogenase, glycerol dehydrogenase, histidinol dehydrogenase and retinol dehydrogenase. Alcohol dehydrogenases generally catalyze NAD(P)(H) dependent reversible oxidation and reduction reactions depending on the available substrates, thus the selected *ADH* genes were supposed to involved in the reversible reactions of furan aldehydes to furan alcohols and vise verse.

Totally 21 *AKR*/*ARI* genes screened from *A. resinae* ZN1 were hypothesized to be responsible for furfural and HMF degradation, including 12 genes encoding aldo–keto reductases and 9 genes encoding aldehyde reductases. Four of these genes were annotated as alcohol dehydrogenases as well: Arz_10923_T1 and Arz_7295_T1 possessed aryl-alcohol dehydrogenase activity, Arz_13663_T1 and Arz_14857_T1 possessed Zn-dependent alcohol dehydrogenase activity. Similar to *ADH* genes, *AKR*/*ARI* genes are also responsible for the dual functions of reduction of furan aldehydes and oxidation of furan alcohols.

Totally 20 *ALDH* genes were identified to be responsible for the conversion of furan aldehydes into the corresponding carboxylic acid forms, including 15 genes encoding NAD-dependent aldehyde dehydrogenase, and 5 genes encoding other aldehyde dehydrogenases including aminoadipate-semialdehyde dehydrogenase, salicylaldehyde dehydrogenase, and betaine aldehyde dehydrogenase.

Totally 16 oxidase genes were hypothesized to possess oxidation function of furan alcohols to the corresponding furan aldehydes and acids with free oxygen as the substrate, including 10 genes acting on the CH–OH group responsible for the conversion of furan alcohols compounds into aldehyde forms, six genes acting on the aldehyde or oxo group involved in the oxidation of furan aldehydes to furan acids.

### Transcriptional quantification in response to furfural and HMF

**Fig. 4:**
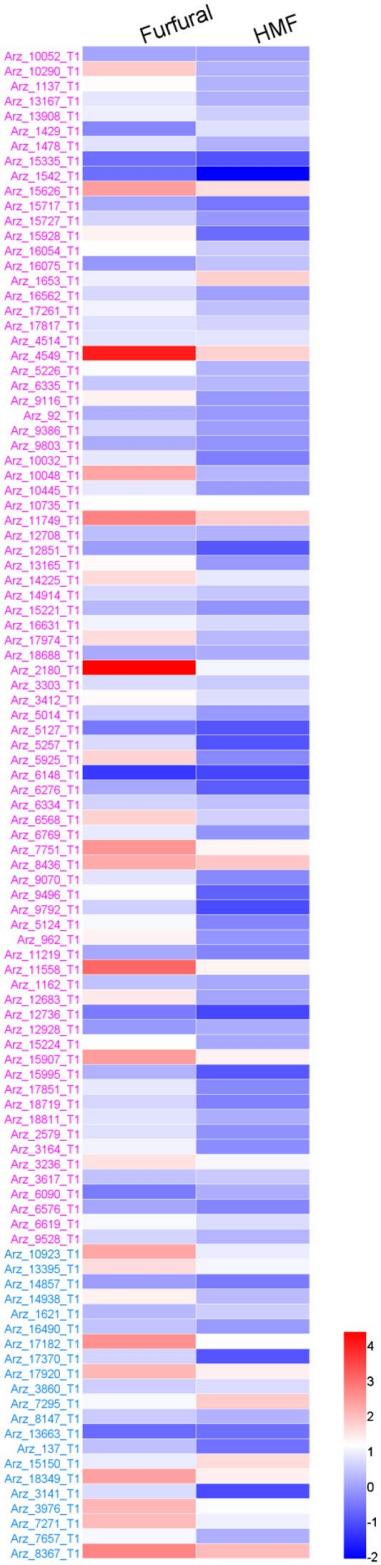
Comparison of transcription levels for selected relevant genes in *A. resinae* ZN1 in response to 1 g/L of furfural or HMF. Quantitative expression level for each gene is log_2_ transformed from raw fold changes against that at 0 h. *Red* indicates up-regulated expression and *blue* for down-regulated expression as indicated by a *color bar* at the *figure right*. The different relevant genes are listed on the *figure left* and the color from *top* to *bottom* indicates different categories of genes: *purple* *ADH* genes, *blue* *AKR*/*ARI* genes

The primary step for inhibitor detoxification in *A. resinae* ZN1 is the fast degradation of furfural or HMF into the corresponding furfuryl alcohol or HMF alcohol to lessen the toxicity on its growth and metabolism [25, 26]. The transcription performance of the 101 genes in *A. resinae* ZN1 on the reduction of furfural or HMF to furfuryl alcohol or HMF alcohol, including 80 *ADH* genes and 21 *AKR*/*ARI* genes, were quantified using qRT-PCR as shown in Fig. 4. Under the stress of furfural, 38 *ADH* genes and 12 *AKR*/*ARI* genes among the total 101 genes were up-regulated by more than twofold, and 1 *ADH* gene was down-regulated. Under the stress of HMF, 12 *ADH* genes and 10 *AKR*/*ARI* genes were up-regulated, 3 *ADH* genes were down-regulated. All the 22 up-regulated genes in response to HMF stress were included in the 50 up-regulated genes in response to furfural, indicating that the 22 genes with the significant enhanced transcription levels were shared for both furfural and HMF reduction (Table 2).

**Table 2:** Genes up-regulated involved in both furfural and HMF degradation in *A. resinae* ZN1

Genes	Genes ID	Functional annotation	Fold change	
			Furfural	HMF
ADH	Arz_15626_T1	Zn-dependent alcohol dehydrogenase, NAD-dependent alcohol dehydrogenase	5.84 ± 3.95	3.33 ± 0.57
	Arz_4549_T1	Zn-dependent alcohol dehydrogenase, NADP-dependent alcohol dehydrogenase	17.75 ± 2.94	3.67 ± 0.16
	Arz_1653_T1	Zn-dependent alcohol dehydrogenase	2.15 ± 1.10	3.71 ± 0.86
	Arz_10735_T1	Short-chain dehydrogenase, levodione reductase	2.48 ± 0.41	2.45 ± 1.23
	Arz_11749_T1	Short-chain dehydrogenase	7.32 ± 3.48	3.81 ± 0.04
	Arz_14225_T1	Short-chain dehydrogenase, 3-oxoacyl-[acyl-carrier-protein] reductase	3.42 ± 0.15	2.02 ± 0.18
	Arz_2180_T1	Short-chain dehydrogenase, 2-(R)-hydroxypropyl-CoM dehydrogenase	22.84 ± 2.24	2.27 ± 0.13
	Arz_7751_T1	Short-chain dehydrogenase	6.23 ± 2.04	2.66 ± 0.26
	Arz_8436_T1	Short-chain dehydrogenase, gluconate 5-dehydrogenase	5.19 ± 0.33	4.05 ± 0.46
	Arz_11558_T1	(+)-neomenthol dehydrogenase	9.38 ± 2.41	2.73 ± 0.11
	Arz_15907_T1	Retinol dehydrogenase	5.87 ± 2.33	2.79 ± 0.44
	Arz_3236_T1	Histidinol dehydrogenase	3.19 ± 0.31	2.30 ± 0.74
AKR/ARI	Arz_10923_T1	Aldo–keto reductase, putative aryl-alcohol dehydrogenase	5.31 ± 0.60	2.09 ± 0.08
	Arz_7295_T1	Aldo–keto reductase, putative aryl-alcohol dehydrogenase	2.32 ± 0.83	3.84 ± 2.51
	Arz_13395_T1	Aldo–keto reductase	3.36 ± 0.03	2.28 ± 0.62
	Arz_17920_T1	Aldo–keto reductase	4.60 ± 2.56	2.82 ± 0.23
	Arz_17182_T1	Aldo–keto reductase, norsolorinic acid reductase	6.44 ± 1.59	2.50 ± 0.93
	Arz_15150_T1	NADPH-dependent methylglyoxal reductase	2.08 ± 0.40	3.34 ± 0.16
	Arz_18349_T1	Aflatoxin B1 aldehyde reductase	5.65 ± 1.13	2.81 ± 0.53
	Arz_3976_T1	Pyridoxal reductase	4.64 ± 1.60	2.42 ± 0.24
	Arz_8367_T1	Pyridoxal reductase	7.12 ± 2.06	4.47 ± 0.31
	Arz_7271_T1	NAD/NADP-dependent indole-3-acetaldehyde reductase	4.48 ± 0.48	2.17 ± 0.43

According to the functional annotation of the *A*. *resinae* ZN1 genome, the 22 up-regulated genes included two Zn-dependent alcohol dehydrogenase genes: the NAD-dependent alcohol dehydrogenase gene Arz_15626_T1 and the NADP-dependent alcohol dehydrogenase gene Arz_4549_T1, similar to the furfural reductase gene (*FurX*) in *Cupriavidus necator* JMP134 [16]; six short-chain dehydrogenase genes: Arz_10735_T1, Arz_11749_T1, Arz_14225_T1, Arz_2180_T1, Arz_7751_T1 and Arz_8436_T1, similar to that in *Clostridium beijerinckii* [27]; two aryl-alcohol dehydrogenases genes: Arz_10923_T1 and Arz_7295_T1, homologous to the aryl-alcohol dehydrogenase genes (*AAD4*, *AAD14*) in *S. cerevisiae* [28]; one methylglyoxal reductase gene Arz_15150_T1, homologous to *GRE2* in *S. cerevisiae* [29]; two pyridoxal reductase genes: Arz_3976_T1 and Arz_8367_T1, which were not reported involved in the degradation of furfural or HMF. The remaining up-regulated genes under the stress of furfural and HMF are three *ADH* genes (Arz_11558_T1, Arz_15907_T1, Arz_3236_T1), three *AKR* genes (Arz_13395_T1, Arz_17920_T1, Arz_17182_T1) and two *ARI* genes (Arz_18349_T1, Arz_7271_T1).

The transcription quantification results indicate that multiple redundant, nonspecific oxidoreductases are responsible for the conversion of furan aldehydes into furan alcohols in *A. resinae* ZN1. All the up-regulated genes in response to HMF stress showed the positive response to furfural stress, indicating that furfural and HMF may trigger similar transcriptional response of oxidoreductases genes to degrade the furan aldehydes into corresponding alcohols. Among these genes, two Zn-dependent alcohol dehydrogenase genes (Arz_15626_T1, Arz_4549_T1) and five *AKR*/*ARI* genes (Arz_10923_T1, Arz_7295_T1, Arz_15150_T1, Arz_3976_T1, and Arz_8367_T1) were significantly up-regulated under the stress of both furfural and HMF, indicating the genes acting on the CH-OH group took the important roles in the degradation of furfural or HMF into furfuryl alcohol or HMF alcohol.

### Transcriptional quantification in response to furfuryl alcohol and HMF alcohol

**Fig. 5:**
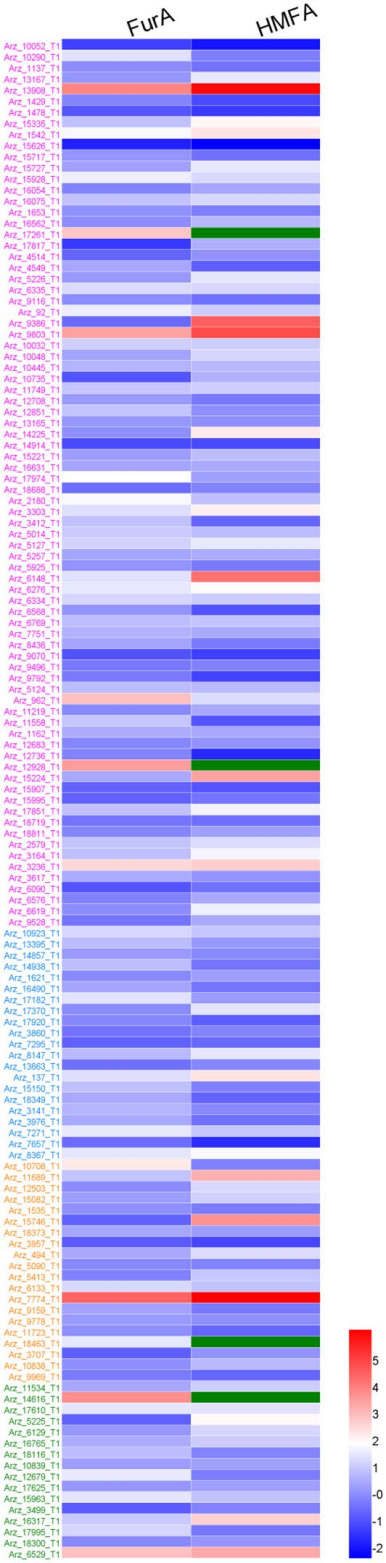
Comparison of transcription levels for selected relevant genes in *A. resinae* ZN1 in response to 1 g/L of furfuryl alcohol or HMF alcohol. Quantitative expression level for each gene is log_2_ transformed from raw fold changes against that at 0 h. *Red* indicates up-regulated expression and *blue* for down-regulated expression as indicated by a *color bar* at the *figure right*. Expression data marked with *green* indicate significantly up-regulated (more than 200-fold) data. The different relevant genes are listed on the *figure left* and the color from *top* to *bottom* indicates different categories of genes: *purple* *ADH* genes, *blue* *AKR*/*ARI* genes, *orange* *ALDH* genes, *green* oxidase genes

*Amorphotheca resinae* ZN1 degrades furfural or HMF into furfuryl alcohol or HMF alcohol, and further into furoic acid or HMF acid under aerobic condition. Furoic acid and HMF acid are commonly considered as almost non-toxic thus the biodetoxification ends with the furan acids formation [26, 30]. However, the degradation mechanism of furan alcohols to furan aldehydes and acids has not been clarified, although furfuryl alcohol and HMF alcohol are less toxic than furan aldehydes and still considered as inhibitors on the cell growth and metabolism of fermenting strains. In this study, the transcription response of *A. resinae* ZN1 to furfuryl alcohol and HMF alcohol were quantified using qRT-PCR. The RNA samples were collected after cultured for 48 h under the stress of furfuryl alcohol or HMF alcohol, respectively, at the time point the degradation of the alcohols was clearly observed. The transcription levels of 80 *ADH* genes, 21 *AKR*/*ARI* genes on the oxidation pathways of furan alcohols to furan aldehydes, as well as 20 *ALDH* genes, 16 oxidase genes involved in the conversion of furan aldehydes to furan acids, were examined as shown in Fig. 5. Under the stress of furfuryl alcohol, 29 *ADH* genes, 5 *AKR*/*ARI* genes, 5 *ALDH* genes and 7 oxidase genes were up-regulated by more than twofold, with only 3 *ADH* gene were down-regulated with the fold change less than 0.5. Under the stress of HMF alcohol, 30 *ADH* genes, 6 *AKR*/*ARI* genes, 8 *ALDH* genes and 8 oxidase genes were up-regulated, with 6 *ADH* genes, 1 *AKR*/*ARI* genes, and 1 *ALDH* genes were down-regulated. The total number of the up-regulated genes under furfuryl alcohol stress was close to that under HMF alcohol stress (46:52).

Different from the commonly shared up-regulated genes under the stress of furfural and HMF, only partial up-regulated genes under furfuryl alcohol stress was shared with that under HMF alcohol stress. However, the ten mostly up-regulated genes with the fold change greater than six under the stress of furfuryl alcohol showed the same significant regulation under the stress of HMF alcohol as shown in Table 3.

**Table 3:** Genes up-regulated more than sixfold involved in furfuryl alcohol and (or) HMF alcohol degradation in *A. resinae* ZN1

Genes	Genes ID	Functional annotation	Fold change	
			Furfuryl alcohol	HMF alcohol
Furan alcohols to furan aldehydes				
ADH	*Arz_13908_T1*	Zn-dependent alcohol dehydrogenases, propanol-preferring	16.93 ± 2.23	70.66 ± 6.23
	*Arz_17261_T1* ^a^	Zn-dependent alcohol dehydrogenases, propanol-preferring	7.93 ± 2.52	718.50 ± 112.23
	*Arz_9803_T1*	Zn-dependent alcohol dehydrogenases, propanol-preferring	12.15 ± 1.19	33.46 ± 3.60
	Arz_9386_T1	Zn-dependent dehydrogenases, diacetyl reductase	–	26.21 ± 3.71
	Arz_6148_T1	Short-chain dehydrogenase, 3-oxoacyl-[acyl-carrier-protein] reductase	–	21.55 ± 4.30
	Arz_962_T1	Iron-dependent alcohol dehydrogenase, hydroxyacid-oxoacid transhydrogenase	8.58 ± 0.42	–
	*Arz_12928_T1* ^a^	Butanediol dehydrogenase/diacetyl reductase	12.88 ± 3.19	208.74 ± 35.63
	Arz_15224_T1	D-arabinitol 2-dehydrogenase	–	12.22 ± 0.60
	*Arz_3236_T1*	Histidinol dehydrogenase	6.50 ± 1.89	7.27 ± 1.35
Furan aldehydes to furan acids				
ALDH	Arz_11689_T1	NAD-dependent aldehyde dehydrogenase, succinate-semialdehyde dehydrogenase	–	10.30 ± 0.15
	Arz_15746_T1	NAD-dependent aldehyde dehydrogenase, succinate-semialdehyde dehydrogenase	–	14.82 ± 1.67
	*Arz_7774_T1*	NAD-dependent aldehyde dehydrogenases	24.61 ± 5.15	78.80 ± 0.77
	Arz_18463_T1^a^	Salicylaldehyde dehydrogenase	–	508.57 ± 14.95
Oxidase	*Arz_14616_T1* ^a^	Glucose-methanol-choline oxidoreductase, alcohol oxidase	15.10 ± 2.07	901.83 ± 192.99
	Arz_16317_T1	Ent-kaurene oxidase	–	7.02 ± 0.93
	*Arz_6529_T1*	Ent-kaurene oxidase	8.54 ± 0.13	11.20 ± 0.27

Italic genes are up-regulated (more than sixfold) response to both two inhibitors, furfuryl alcohol and HMF alcohol

^a^Genes are significantly up-regulated (more than 200-fold) involved in HMF alcohol degradation

– Relative expression means not up-regulated or up-regulated to relative lower level (less than sixfold)

Under the furfuryl alcohol or HMF alcohol stress, the propanol-preferring Zn-dependent alcohol dehydrogenases genes (Arz_13908_T1, Arz_17261_T1, and Arz_9803_T1) were significantly up-regulated, in which Arz_17261_T1 was up-regulated more than 700-folds at 48 h under the HMF alcohol stress. Histidinol dehydrogenase gene Arz_3236_T1 and the butanediol dehydrogenase gene Arz_12928_T1 were also up-regulated in response to the stress of the two alcohols. The other significantly up-regulated *ADH* genes response to HMF alcohol stress only included diacetyl reductase gene Arz_9386_T1 (26.21-fold), 3-oxoacyl reductase gene Arz_6148_T1 (21.55-fold), and arabinitol 2-dehydrogenase gene Arz_15224_T1 (12.22-fold).

The NAD-dependent aldehyde dehydrogenase gene Arz_7774_T1 was up-regulated by 24.61- and 78.80-fold, respectively, under the furfuryl alcohol and HMF alcohol stress. The other up-regulated *ALDH* genes in response to HMF alcohol stress also included Arz_11689_T1 and Arz_15746_T1 encoding the NAD-dependent succinate-semialdehyde dehydrogenases, Arz_18463_T1 encoding salicylaldehyde dehydrogenase with broad substrate specificity accepting mono- and di-aromatic aldehydes but not aliphatic aldehydes [31].

Two genes among the 16 selected oxidases genes, Arz_14616_T1 and Arz_6529_T1, were significantly up-regulated in response to both furfuryl alcohol and HMF alcohol stress. Arz_14616_T1 encodes a glucose-methanol-choline oxidoreductase, similar to HmfH in *C*. *basilensis* HMF14 responsible for furfural or HMF to its corresponding acid form [20]. Arz_6529_T1 encodes a multifunctional ent-kaurene oxidase catalyzing alcohols to aldehydes, or aldehydes to acids with NADH or NADPH as one donor and incorporating one atom of oxygen. Moreover, Arz_16317_T1 (7.02-fold), another up-regulated gene in response to HMF alcohol stress, encodes the same protein with Arz_6529_T1.

The transcription analysis under the stress of furfuryl alcohol or HMF alcohol reveals that the conversion of furan alcohols to furan aldehydes and furan acids was catalyzed by the propanol-preferring Zn-dependent alcohol dehydrogenase genes (Arz_17261_T1, Arz_9803_T1 and Arz_13908_T1) and the NAD-dependent aldehyde dehydrogenase gene (Arz_7774_T1), respectively, or by the oxidase genes (Arz_14616_T1 and Arz_6529_T1) with oxygen as the acceptor. Based on the results, the role of oxygen in the further degradation of furan alcohols to furan acids as observed in different air input conditions in our previous work [24] was concluded at the molecular level. There are two possibilities, one is the highly reduced redox state under aerobic condition compared to under anaerobic condition, which facilitates furan alcohols converted into furan aldehydes and acids by NAD(P)^+^-dependent oxidoreductases. The second possibility is the function of the oxidases. Under aerobic condition, *A*. *resinae* ZN1 can trigger the regulation for transcription response of corresponding oxidase genes and these oxidase proteins with oxygen as the substrate can instead of those nonspecific, redox power-depending oxidoreductases to promote the further degradation of furan alcohols to furan aldehydes and acids.

Table 3 also shows that the HMF alcohol stress initiated hundreds-fold up-regulated expression of two *ADH* genes (Arz_17261_T1, Arz_12928_T1), one *ALDH* gene (Arz_18463_T1) and one oxidase gene (Arz_14616_T1), which is much higher than that under the furfuryl alcohol stress. However, the actual degradation rate of HMF alcohol is lower than that of furfuryl alcohol, indicating the specificity of these enzymes to HMF alcohol were not as high as to furfuryl alcohol and higher expression of the enzymes are required for HMF alcohol degradation.

## Conclusions

The degradation mechanism of furfural and HMF reduction to the corresponding alcohols, as well as the alcohols oxidation to the corresponding aldehydes and acids in *A. resinae* ZN1 was investigated using real-time qPCR method elaborating a complete picture of furfural and HMF biodegradation at the molecular level. The genes involved in the degradation metabolism of furan aldehydes into the less toxic compound furan alcohols and furan acids were identified. Zn-dependent alcohol dehydrogenases and aldo–keto reductases with NAD(P)H as the cofactor were up-regulated for the degradation of furfural or HMF into corresponding furfuryl alcohol or HMF alcohol. Propanol-preferring alcohol dehydrogenases, NAD(P)^+^-dependent aldehyde dehydrogenases, or oxidases with free oxygen as the acceptor were up-regulated for the oxidation of furfuryl alcohol or HMF alcohol into its aldehyde and acid form under aerobic condition. This study provided us efforts in constructing more robust strains for efficient lignocellulosic ethanol production.

## Methods

### Strains, media, and culture conditions

Biodegradation fungus strain *A. resinae* ZN1 was isolated in our previous work and stored in China General Microbiological Culture Collection Center (CGMCC), Beijing, China with the designation number of CGMCC 7452. *A. resinae* ZN1 was cultured in either potato-dextrose-agar (PDA) medium containing 200 g/L of potato extract juice, 20 g/L of glucose with 15 g/L of agar, or synthetic complete medium containing 2 g/L of KH_2_PO_4_, 1 g/L of (NH_4_)_2_SO_4_, 1 g/L of MgSO_4_·7H_2_O, 0.5 g/L of CaCl_2_, and 1 g/L of yeast extract with or without glucose according to the experimental design. The fungus was grown at 28 °C in flask without agitation. Two replicated experiments were carried out for each culture.

The whole genome of *A. resinae* ZN1 has been sequenced and deposited in GenBank under accession number of JZSE00000000. Total 18,830 protein-coding genes were predicted and functioned annotated using a series of database including KEGG (Kyoto Encyclopedia of Genes and Genomes), KOG (Eukaryote clusters of orthologous groups), Swiss-Prot and NR (the non-redundant protein database).

### Cell growth and metabolic response of furfural and HMF

*Amorphotheca resinae* ZN1 was maintained from a lyophilized stock and on PDA slants at 4 °C. Spores were harvested by washing each PDA slants with 20 mL of the deionized and sterilized water. Then 10 % of the spore suspension (4-5 × 10^6^ spores/mL) was inoculated into the synthetic complete medium containing 20 g/L of glucose and incubated at 28 °C. The periodic sampling of *A. resinae* ZN1 broth from the fermentor was not allowed to measure the cell mass accurately because of the flocculation of cell mycelia and aggregation [24]. In this study, *A. resinae* ZN1 was incubated in 100 mL conical flasks, and the cell growth was measured by weighting the whole dry cell weight of the flasks.

When furfural or HMF was used as the carbon source, the seed culture was carried out for 6 days, and then the cell mycelium was harvested by washing with sterile ddH_2_O twice to remove the residual glucose, and suspended in the same volume of inorganic salt medium (synthetic complete medium without yeast extract addition). 3 mL of the seed suspension was transferred to 30 mL of the fresh inorganic salt medium containing 1 g/L furfural or HMF in 100 mL shake flask. When glucose was used as the carbon source, the inoculum culture was grown for 4 days, then the whole cell mass was dispersed and directly inoculated into 30 mL of the synthetic complete medium containing 5 g/L glucose and 1 g/L of furfural or HMF. One flask was taken out for cell mass analysis at the specific time intervals continuously till the end of each culture. The dry cell weight (DCW) was obtained by filtrating 30 mL of the culture broth into the cell pellet, and dried at 105 °C for 12 h until constant weight. At the same time, 1 mL of the supernatants were collected and stored at 4 °C until used for glucose and inhibitors concentration detection.

### Analytical methods

Residual glucose in the medium was monitored with a SBA-40D glucose analyzer (Shandong Academy of Sciences, Jinan, China).Furan compounds were analyzed using reverse-phase HPLC (LC-20AT, SPD-20A UV detector, Shimadzu, Kyoto, Japan) with a YMC-Pack ODS-A column (YMC, Tokyo, Japan) at the column temperature of 35 °C [24]. Furfural, furfuryl alcohol, and furoic acid were analyzed using 50 % acetonitrile solution as the mobile phase at the rate of 1.0 ml/min and the detection wavelength of 220 nm. HMF, HMF alcohol, and HMF acid were analyzed at the mobile phase rate of 0.6 mL/min, and the detector wavelength was 230 nm. The gradient procedure applied pure water as the solvent A, acetonitrile as the solvent B, and the initial flow phase was at a ratio of 95 % (A) to 5 % (B): firstly, acetonitrile was increased from 5 to 100 % over 0–15 min; then acetonitrile was decreased from 100–5 % over 15–20 min; finally, acetonitrile was used at 5 % over 20–30 min. All the samples were filtered through a 0.22-μm filter before analysis.

### Samples collection, RNA extraction, and qRT-PCR

When the furan inhibitors was used as the sole carbon source, the cell mass of *A. resinae* ZN1 was too low for RNA extraction and transcriptional analysis. Therefore, cells used for gene expression analysis were cultured with 5 g/L of glucose addition. When the transcription of *A. resinae* ZN1 under the stress of furfuryl alcohol or HMF alcohol was examined, the cells were cultured without glucose addition. The detailed operation for cell mass collection was described as follows.

When furan aldehydes were added, seed culture of *A. resinae* ZN1 was inoculated into the fresh synthetic complete medium with 5 g/L glucose and precultured for 2 days at 28 °C. When the hyphae were formed in the culture medium, furfural or HMF was added to reach 1 g/L as the starting point for cell mass collection. After 4 h, the cell mass was collected again.

When furan alcohols were added, seed culture was transferred to synthetic complete medium without glucose addition. After preculture for 2 days, 1 g/L of furfuryl alcohol or HMF alcohol was added and cell mass was harvested at 0 and 48 h after inhibitor treatment by centrifugation at 12,000 rpm for 10 min at 4 °C, snap frozen in liquid nitrogen and then stored at −80 °C for subsequent analysis.

The total RNA was extracted using Trizol reagent (RNAiso Plus, TAKARA, Otsu, Japan) after the cell mass was grinded. The RNA integrity was assessed by gel electrophoresis and the RNA quantity was determined by DU 800 spectrophotometer (Beckman Coulter, Fullerton, CA, USA). Reverse transcription reactions were carried out using ReverTra Ace qPCR RT Master Mix with gDNA Remover kit (TOYOBO, Osaka, Japan) according to the manufacturer’s protocol. For each qRT-PCR reaction, a SYBR Green Realtime PCR Master Mix kit (TOYOBO, Osaka, Japan) was used and the PCR reaction was run on a BioRad CFX 96 system (BioRad, Hercules, CA, USA).

The β-actin gene Arz_9569_T1 of *A. resinae* ZN1 was served as an internal control to normalize for difference in total RNA quantity. Transcription levels of each interesting genes were quantified using the formula 2^−ΔΔCt^. Differentially expressed genes were determined with a selection threshold of fold change ≥2.0 (up-regulation) or ≤0.5 (down-regulation).

The optimized 18–25 bp primers for qRT-PCR analysis were listed in the Additional file 1: Table S1. The primers were designed using Primer Premier 5 software based on the total genome sequence of *A. resinae* ZN1. The amplification length of all test genes ranged from 120 to 170 bp. In addition, the amplification efficiencies of each amplicons were tested by performing qPCR with a serial-diluted cDNA as the template, and only those whose being between 90 and 110 % were used for gene expression analysis. qPCR reactions were set up in a total volume of 20 μL consisting of 2 μL of cDNA (diluted 1: 4), 2× SYBR Green Mix (TOYOBO, Osaka, Japan), 6.4 μL of ddH_2_O and 0.8 μL of gene-specific primers. PCR conditions were as follows: 95 °C for 1 min; 40 cycles of 95 °C for 15 s, 55 °C for 15 s and 72 °C for 30 s; a final melting curve step by heating from 65 to 95 °C with a speed of 0.5 °C per 5 s.

## Additional file

Additional file 1:
**Table S1.** Sequences of primers used for transcription and expression analysis by real-time qRT-PCR using SYBR Green.

## Abbreviations

ADH: alcohol dehydrogenase
AKR: aldo–keto reductase
ARI: aldehyde reductase
ALDH: aldehyde reductase
CGMCC: China General Microbiological Culture Collection Center
CoA: coenzyme-A
DCW: dry cell weight
EMP pathway: glycolysis pathway
FDCA: 2,5-furan dicarboxylic acid
HMF: 5-hydroxymethylfurfural
HMF alcohol: 5-hydroxymethylfurfuryl alcohol
HMF acid: 5-hydroxymethylfuroic acid
KEGG: Kyoto Encyclopedia of Genes and Genomes
KOG: eukaryote clusters of orthologous groups
NADH: nicotinamide adenine dinucleotide (reduced)
NADPH: nicotinamide adenine dinucleotide phosphate (reduced)
NAD: nicotinamide-adenine dinucleotide
NADP: nicotinamide adenine dinucleotide phosphate
qRT-PCR: real-time quantitative polymerase chain reaction
PDA: potato dextrose agar medium
TCA cycle: tricarboxylic acid cycle
