# Supplementary material for: Transcriptional analysis of Amorphotheca resinae ZN1 on biological degradation of furfural and 5-hydroxymethylfurfural derived from lignocellulose pretreatment
Source: Biotechnol Biofuels. 2015 Sep 4;8:136. doi: 10.1186/s13068-015-0323-y (PMC4559888; doi:10.1186/s13068-015-0323-y)
Supplement: Additional file 1: — Table S1. Sequences of primers used for transcription and expression analysis by real-time qRT-PCR using SYBR Green. [file 13068_2015_323_MOESM1_ESM.docx]

**Table S1.** **Sequences of primers used for transcription and expression analysis by real-time qRT-PCR using SYBR Green.**

| Gene ID | Primers sequence 5’-3’ | |
| --- | --- | --- |
|  | Forward | Reverse |
| **ADH** |  |  |
| Arz_10052_T1 | gccagacatcaaatccatgagc | tcgccatcaagtcactaccaaac |
| Arz_10290_T1 | gccaaaggaacctaccaacg | accagccgcaacctcaatc |
| Arz_1137_T1 | gcagattgcgaaagccagtg | ctcgtcgtccgtcatcttcag |
| Arz_13167_T1 | acccgcaggaggaagatgtg | gtatcccacttggcaacattcac |
| Arz_13908_T1 | aacactggtttgcgttggtatg | tcagatgggtcttcacgatgc |
| Arz_1429_T1 | accgttccttacctgccaatca | catggggaactcttcgatgatg |
| Arz_1478_T1 | gggcttatctcaattattggattcc | cctcaatggctctgttcatctcc |
| Arz_15335_T1 | tggagggatggtggatagaag | tccaggatcactaggcacgtac |
| Arz_1542_T1 | tggagattggattacctcaagtcc | agtaatgagcccagccctttg |
| Arz_15626_T1 | cagcagttgaagtagcctccg | gaatttcttcagactcgcaagatc |
| Arz_15717_T1 | ttaggttgctggcgggtaaagac | gcttcaataaccaccttcaacacca |
| Arz_15727_T1 | agccctgatctcaagaaagcc | ccgacgccaacagtaaccac |
| Arz_15928_T1 | gattggtgaagagggtaaaggagc | cagggaatctcctcctggtgaac |
| Arz_16054_T1 | gcttgtcccattgttatcaccg | ttccacaatagccttgcctgc |
| Arz_16075_T1 | gacatcacgttccccatcatg | gcccgtaatcaacttcttgacat |
| Arz_1653_T1 | ggcttcgtgttattgggtccg | catttggtgccaggcgagtc |
| Arz_16562_T1 | atggataggcattgttggctctg | gcaccagcttccttcgataactc |
| Arz_17261_T1 | cgtcgcccattccagtcatac | ccttggcaccctctatttcctct |
| Arz_17817_T1 | ggcagtggatcggtatcattgg | tccctatcctcgcatcaagcac |
| Arz_4514_T1 | ttcgcattacggggaacctc | aactatcctgcccgcaatgtc |
| Arz_4549_T1 | atcgtcggtattggaggtcttg | catctgtggcaatgaatctgtcc |
| Arz_5226_T1 | attgaaggaaacaaagcacgtc | gaccaagttcacacctcgctc |
| Arz_6335_T1 | tgtactggagttgaatcttgccttc | gcatcatcgtatgtatttgcgtatc |
| Arz_9116_T1 | accgcatgacgtttgacct | caatcagacatccattcgctat |
| Arz_92_T1 | caggagaagctgatcgagatgac | ctgcgacaccaatgacgatg |
| Arz_9386_T1 | tacgtgcggcaggatttcg | gacatgcttctccttctccgtg |
| Arz_9803_T1 | tgacaccgctgaggctattga | ttccagcatgagatcgtacacct |
| Arz_10032_T1 | attcgtgtgaactccatttccc | cacttgccaaaaacacacatcc |
| Arz_10048_T1 | tcgttgagaaagatgagtgtagcg | cgaatgtcctggtccagatgg |
| Arz_10445_T1 | agaagtacccacccaacctcc | ggaatcaggcagtccaccag |
| Arz_10735_T1 | ttgccaaagagcgcagattag | ggttaatattgttgatggcaagatg |
| Arz_11749_T1 | tctttgagagagcgtgaacctgac | atctggcaatccgagaggtagg |
| Arz_12708_T1 | gaaaggagcgatgtgaaagacg | tctcgtcgtgctgctggttc |
| Arz_12851_T1 | tagctgctgagaacccgaatg | gagccagaggatgaagtgtgc |
| Arz_13165_T1 | cgttatctcaggagaggtgtctacc | gtcatagcgtcaggacttctgttg |
| Arz_14225_T1 | gcaggcattgtggctattcg | gttcgtacaacaccgggacag |
| Arz_14914_T1 | cacatcaaggatgtcacggagg | cctttgcttgacccgtaatgc |
| Arz_15221_T1 | cgtgtacttggtgacgagggc | aagcgttcttgctggacttgtag |
| Arz_16631_T1 | cccaacccaccaagaacatc | gggagacagtatccaccatcaac |
| Arz_17974_T1 | attctacggcgacaagtcaaagg | ggttcgggaagatcagagtatcag |
| Arz_18688_T1 | agatcgtggcggaactgaag | cgccatgacgatcaacagag |
| Arz_2180_T1 | tttggacgattggactactgtgtg | gccaacatagccctaatctgtgc |
| Arz_3303_T1 | gctggaacaggcgttcgtatc | gccactcttgcgacctcatc |
| Arz_3412_T1 | ccgaccatttactcggatgc | cttccacgacggaatacttcg |
| Arz_5014_T1 | tcttgggaaacggaatattacgtc | tatatcctgagggcttcctatcctc |
| Arz_5127_T1 | ggcattggaagagccgtagc | tctgagcactccgcaaatcg |
| Arz_5257_T1 | tggcaacatcctcctatccg | gacatccatttccccattcg |
| Arz_5925_T1 | atcttctgctccagcgttgc | ccattgaccgtgatgccag |
| Arz_6148_T1 | ggtgaaagcggagagaaccag | accgtctgcccattcaggtac |
| Arz_6276_T1 | tatcatcacggcatccatagcag | tgtgtcgtggacccaattcaac |
| Arz_6334_T1 | gagactgaggagtatcagtgagcg | catccaaccaccatcgacaac |
| Arz_6568_T1 | tgtcggctagtagtttcgcctatg | acctgctgcgagatcggatg |
| Arz_6769_T1 | gtcagtttgccgagttcctagac | cgagatggatgagatggctatg |
| Arz_7751_T1 | gagtctggcgagggagtttg | gatcgtggctgtgtgtgtagatg |
| Arz_8436_T1 | tcaacatcacgtctggcttgg | ctctctctcgatccgatcattctc |
| Arz_9070_T1 | catgatgatagcgaccaatcttaac | cattgtacgcaaagccgtataag |
| Arz_9496_T1 | cctagattcgcaagctacgagc | cccaccgtcgacattgagtatc |
| Arz_9792_T1 | cggatatgctagatgaggtagtcg | ggtatggcgttaagggcttc |
| Arz_5124_T1 | gctgtggtcagcgagttaggtc | ccagggatctttcaagcagttg |
| Arz_962_T1 | gcttcaagagagaaaacatcgacg | tgttgtcgctcttcattcacctc |
| Arz_11219_T1 | gcactccaccatcaaggttaagg | ttgtttccaagaccacgagcac |
| Arz_11558_T1 | ttgctcttgtcatactcgcacc | gccagtctgaccgcattaatag |
| Arz_1162_T1 | cgatgctctcgtcatggaaac | aatcctccagttcctcgctcac |
| Arz_12683_T1 | cgagatacggcatgagtaaattg | cttgggtggggtttgatttg |
| Arz_12736_T1 | tgccggttatgcacagtcc | aactggatcttggaagtgtttctg |
| Arz_12928_T1 | gctagtatcgtggcattcaaacc | caataaggtcccacatcgcag |
| Arz_15224_T1 | agcgtcaatgtcacgggatgt | cggctttggaggcgttgtag |
| Arz_15907_T1 | gtgtctactctgaatcctggttgg | aggtactgtccatgcgtttcac |
| Arz_15995_T1 | atcatcactcggattggaaacc | tggaaacggaaagccatctc |
| Arz_17851_T1 | atcaatctcagcggcatcatg | atgacaccgaatttgctcgtc |
| Arz_18719_T1 | caggtgctggatggtcagaag | catcatgtcccaggcgaaac |
| Arz_18811_T1 | acacggtcgagaactggcag | tgacgagatattgatgatagaggc |
| Arz_2579_T1 | ggaacttgtataccgtgaaattgg | tgcttggaggtagcgtagatagg |
| Arz_3164_T1 | cggaatacggcgagaagaac | cccaagtgggacattagatacg |
| Arz_3236_T1 | cagcggatcctttcacagtag | caagaggctctgcacgaaag |
| Arz_3617_T1 | tgtagtagattttccagaccgattg | gctaccatctgaacgaggcg |
| Arz_6090_T1 | gagccatcgtcaatgtcccac | tcagtgctgtaagcatgtaaccg |
| Arz_6576_T1 | aacgcagttgttgagaagtacgg | gccatcatagccttacccacag |
| Arz_6619_T1 | agttgcggaagcataacgatagag | ggctgagcattaacggtgagc |
| Arz_9528_T1 | gccataacaatcctgcccc | gtttccatgacgagagtatcgc |
| **AKR/ARI** |  |  |
| Arz_10923_T1 | cctgatctaccgtgaagaagaacg | gttgttcagcatcctgtccgtc |
| Arz_13395_T1 | gggacattgtgcaatggtgc | agaggcacgaaacccttctgtag |
| Arz_14857_T1 | ttcaacacgcagacggacatc | gcactcgtacttcttggacagc |
| Arz_14938_T1 | agaagattggcggctcagtg | tctttcatcacctcatccgtcag |
| Arz_1621_T1 | tggtatttccaacttccaaggttc | caaaggacgagtatgcggtgac |
| Arz_16490_T1 | acccaactcttgtcgagatcgg | ccctccaagtttgcctttatcc |
| Arz_17182_T1 | ggtaacttcaagtccgacgagc | cgtatgccagagcgacactg |
| Arz_17370_T1 | ctcaagccgattgccgatag | gccttcacgctgtcataaacc |
| Arz_17920_T1 | agtggaccaaggatagcatgac | gactagagcctcgacggtcttc |
| Arz_3860_T1 | tttggaatagcgttttggatgg | cataattctcatctgggctcgg |
| Arz_7295_T1 | aacagactatatcgaccttctccag | ccagccattcttctcagcg |
| Arz_8147_T1 | aggagatcgtggatttctgtaagg | tgataactcaggaggacggagg |
| Arz_13663_T1 | cgagaagaacgacgactacacc | cttcttggtccagttggacacg |
| Arz_137_T1 | ttccgcattaaatatcaaggatgg | ttccgtgtctgtcttgctcacc |
| Arz_15150_T1 | aaggctgcttgggagttcg | tcgctggttggaggtgttg |
| Arz_18349_T1 | cttcccatgccttcgtcattac | gtatcggaccctgtaactttgacc |
| Arz_3141_T1 | cagaacaagccatcatttgcc | gtgccgatgtttggtgagatg |
| Arz_3976_T1 | cgagacgacattgaaggtacttg | gttgcccacagtgagagttcc |
| Arz_7271_T1 | ctacctccaacatggtgatctcg | ctcccttgctgactccgtatttc |
| Arz_7657_T1 | gaccagagtgttgtagctgtgacg | atcttccttcagacaccctgaacc |
| Arz_8367_T1 | aactggtcaacgagggcaag | tgtccaatgcgtgtcgtgag |
| **ALDH** |  |  |
| Arz_10708_T1 | cctcacgacatcccccactatc | cacgcaatagcaccgtttactg |
| Arz_11689_T1 | gaattgcccttttctgatctttg | ggtcgtagatgctgctttggac |
| Arz_12503_T1 | gtgctcggatgggctgaag | gacgcggttggcagtgatg |
| Arz_15082_T1 | gtggaccgagacgctcaaac | aacgaccgctcccgtactct |
| Arz_1535_T1 | ggaacttcgtgaagccaacc | ggtccagatagatgcggtgag |
| Arz_15746_T1 | ttggcactcctgtcattcgac | gttccctgtattcatgccgatc |
| Arz_18373_T1 | gccgacaatcatcgaaacg | gtccagaccgaggctcctaac |
| Arz_3957_T1 | acccaaccgagcatcagaac | aagtagcctttggagttatcgtagg |
| Arz_494_T1 | atggaccgatacgctcaaactc | ccgctcccgtactcttagtattg |
| Arz_5090_T1 | acgagcgagctaaagcccaac | gtctgcggatttctttaagcc |
| Arz_5413_T1 | cgaggaccgcaggtagataag | ctttatcgcccgactgttctc |
| Arz_6133_T1 | tcaacagcagcaacgactcc | attcacatggaccgccttagtc |
| Arz_7774_T1 | acaatgctgctgtgctggttc | cgtattggagttgggagacctg |
| Arz_9159_T1 | cgggtctttgtccagcgag | accttgaggcacggtgagc |
| Arz_9778_T1 | gcaagttgacgtaggagcaatg | agggtcggttggaaatagtgtc |
| Arz_11723_T1 | ggtcgtcgtagcttcagcac | tcttcttccactccgcatactc |
| Arz_18463_T1 | tgaagtgcggaagccaatatg | cgagatcgccagtcaacagc |
| Arz_3707_T1 | ctcttcaaggctgctgtttcg | atgtcgccgtgaattatcgtc |
| Arz_10838_T1 | gccaagatcccgtcaggctac | gcgagtccgagtttcgtgtta |
| Arz_9969_T1 | gaactccaaggggtatgctaagag | tgaagatttcagaccgaggctag |
| **Oxidase** |  |  |
| Arz_11534_T1 | accttcaagatcacggctacg | tgggcataaacgctgatacg |
| Arz_14616_T1 | gttgagactacatggcacagtctg | ccgcaaccttcaggttcttaac |
| Arz_17610_T1 | aatagccgtggaggcgttg | gagattacgtccaatgtgccg |
| Arz_5225_T1 | ccgataaaccactgaccgatg | cccctgggactgcagagaag |
| Arz_6129_T1 | cacaggaaaaccgaggtcaac | tgtgtgccgtagaagacccag |
| Arz_16765_T1 | cgagggaacgtccacataag | tatttcggctgtgaaggcac |
| Arz_18116_T1 | gggagcggatatgcatttgtg | cgtcgtgcaggtaatcaaggag |
| Arz_10839_T1 | gccgacttatgcttctttctg | tccatcgtggtttgaggtg |
| Arz_12679_T1 | actcgtcggtttcccacgta | atggggacgaaatcgatctg |
| Arz_17625_T1 | ctctcggcttgggaaaattct | attgacaagtgtgctggacttactc |
| Arz_15963_T1 | ggagtcacgttgcccgataa | tctgcccattgattcttgcc |
| Arz_3499_T1 | gtcctcatctccggctctga | cgatgttgccttcgttgtagttc |
| Arz_16317_T1 | tgatccagataccttcggtgatg | gccaggacatgcttggctac |
| Arz_17995_T1 | gttgattccgtcatgtctaccg | ggggatagtcacaccgtcgt |
| Arz_18300_T1 | tgttggcttaccgctttgc | gacggagattgatgcgttgg |
| Arz_6529_T1 | atggcaatatggtatggctcag | ggagtgggagtccttgtgaatc |
